# Neoadjuvant radiotherapy of primary irresectable unicentric Castleman's disease: a case report and review of the literature

**DOI:** 10.1186/1748-717X-5-7

**Published:** 2010-02-02

**Authors:** Iris AC de Vries, Marjolein MS van Acht, Thomas BJ Demeyere, Marnix LM Lybeert, Jean-Paul de Zoete, Grard AP Nieuwenhuijzen

**Affiliations:** 1 Department of Surgery, Catharina Hospital, Eindhoven, The Netherlands; 2 Department of Pathology, Catharina Hospital, Eindhoven, The Netherlands; 3 Department of Radiotherapy, Catharina Hospital, Eindhoven, The Netherlands

## Abstract

**Background:**

Castleman disease (CD) is a rare benign disorder characterised by hyperplasia of lymphoid tissue that may develop at a single site or throughout the body. The etiology of this disorder is unclear, although the histopathological presentation can be differentiated into a hyaline vascular variant, a plasma cell variant and a mixed variant. Clinically, it has been recorded that 3 manifestations of CD are characterized: a localized unicentric type, a generalized multicentric type and a mixed form. Surgery remains the main treatment for resectable unicentric CD, since removal of the large node is possible without further complications. No consensus has been reached concerning the most adequate treatment for irresectable unicentric CD.

**Methods:**

Case report of a 67 year old woman.

**Results:**

This report, describes the case of a 67-year-old woman with unicentric Castleman disease located in the right lower abdomen. The patient had symptoms of fatigue, dyspnoea and pain in the right lower abdomen. Computed tomography (CT)- examination revealed a tumour, which had grown to form a close relationship with the common iliac vessels and the sacral bone. A Laparotomy procedure revealed that the tumour was an irresectable mass. Neo-adjuvant radiotherapy (40 Gy) was administered in order to downsize the tumour. Six weeks later a new CT-scan revealed a major reduction of the tumour, which enabled a successful radical resection of the tumour to be performed. Histopathological analysis of the tumour showed the hyaline vascular type of CD.

**Conclusions:**

Neo-adjuvant radiotherapy should be considered in case of an irresectable unicentric CD.

## Background

In 1954, Benjamin Castleman described an unusual benign disorder that was characterized by hyperplasia of lymphoid tissue [1]. A couple of years later, Castleman et al [2] published more cases with benign massive growth of lymph nodes that is commonly referred to as Castleman's disease (CD). Flendrig et al [3] categorised two main types and one mixed variant of CD. Keller et al [4] defined these histopathological different patterns.

The first being a hyaline vascular type (HV), characterized by lymphoid follicular hyperplasia with involuted germinal centres, which are partly or totally replaced by deposit of hyaline material and transfixed by a radially penetrating vessel, characterized as a 'lolly pop' structure. The second variant was defined as the plasma cell type (PC) with a follicular hyperplasia of hyperplastic, large germinal centres in which the interfollicular areas were occupied by large sheets of plasma cells.

Clinically a broad spectrum of manifestations of CD are described, ranging from an asymptomatic localized lymphadenopathy to a severe symptomatic multifocal or generalized lymphadenopathy [5]. A commonly used system to classify the heterogeneity of CD was proposed by McCarty et al in 1995 [6]. This made a distinction between the unicentric and the multicentric forms of disease. This classification correlates quite well with the histopathologically variants. As the HV type is mostly unicentric and the PC type and the mixed variant seem to be mostly multicentric [7,8].

CD has to be treated because of its progressive course associated with local involvement of surrounding structures or because of the systemic effects associated with the multicentric form such as fever, weight loss, excessive sweating, hemolytic anaemia, splenomegaly, oedema and neuropathy.

CD can also be associated with HIV infection, POEMS syndrome, amyloidosis, renal insufficiency and increased risk of lymphoma [5,9-11], these conditions necessitate diagnostic and therapeutic intervention.

Surgery is considered to be the most adequate therapy for unicentric resectable cases of CD, as it seems to be curative in almost all of the cases [4,6,12-20]. Radiotherapy has also been described as a definitive treatment, however with a variable response rate [4,9,10,21-30].

No consensus has been reached concerning the most adequate treatment for irresectable CD. To our knowledge, neo-adjuvant radiotherapy to downsize primary irresectable CD in order to achieve a radical surgical resection has not been described yet. This paper reviews the associated literature concerning the treatment of CD and describes a case history of an irresectable unicentric localisation of CD which was treated with neo-adjuvant radiotherapy and subsequent radical resection.

## Methods

### Case presentation

A 67-year-old woman with a history of hyperthyroidism, proptosis and anaemia was referred to the internal medicine department with complaints of weight loss, fatigue, dyspnea and pain in the right lower abdomen. Physical examination revealed a painful palpable mass in the right lower quadrant of the abdomen.

**Figure 1:**
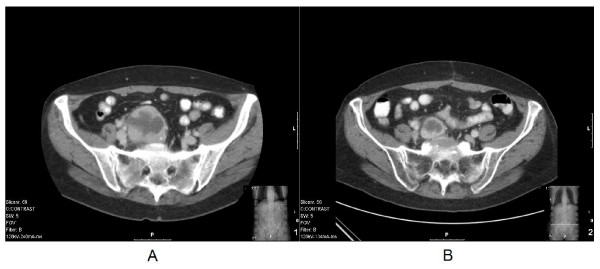
**A: Computed Tomography (CT) of the abdomen showing the tumour and its relationship with the sacral vertebrum, the adipose tissue and the common iliac artery and vein, before neo-adjuvant radiotherapy**. ****B: **Computed Tomography (CT) of the abdomen showing the marked downsizing of the tumour, after neo-adjuvant radiotherapy.**

**Figure 2:**
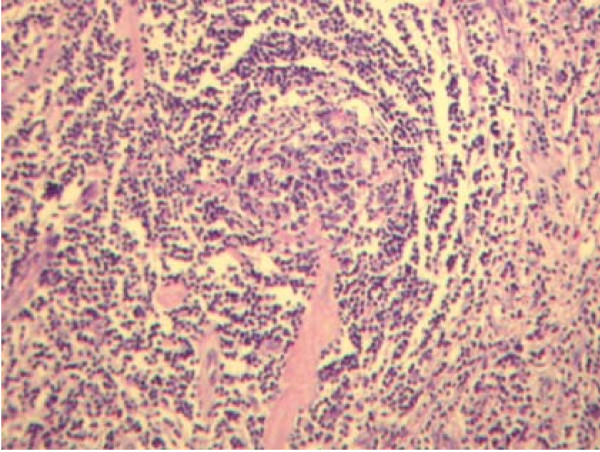
**Histopathology showing lymphoid hyperplasia, atrophic germinal centers and radialy penetrating vessels (lollypop phenomenon) as seen in the HV variant of CD**.

Ultrasonography of the abdomen showed a multicystic, solid structure in the right lower abdomen. Subsequent Computed Tomography (CT) imaging of the abdomen showed right ventrally of the body of the first sacral vertebrum a partly solid, partly cystic tumour of 71 × 51 mm with oedematous infiltration of the surrounding adipose tissue and a close relationship with the common iliac artery and vein (figure 1A). Since the radiological aspect was initially presumed to be indicative of ovarian cancer, the patient was referred for gynaecological examination. Transvaginal ultrasound showed a polycystic structure on the right side. CA125 was normal. The patient underwent a staging laparotomy by the gynaecologist, revealing a normal uterus, normal ovaries and a fixed retroperitoneal tumour located to the right side of the aortic bifurcation. A surgeon oncologist was consulted, and because of the fixation and close relationship of the tumour to the iliac vessels and sacral bone, the tumour was regarded as primary irresectable and only an incisional biopsy was performed to obtain material for histological examination. Histopathology from the specimen revealed lymphoid hyperplasia, atrophic germinal centers and radialy penetrating vessels (lollypop phenomenon) as seen in the HV variant of CD, (figure 2). A subsequent positron emission tomogram (PET)-scan revealed no signs of disseminated disease. There were no laboratory abnormalities and the HIV test turned out to be negative. Since the tumour was regarded irresectable, neo-adjuvant radiotherapy was proposed in order to downsize the tumour to achieve a radical surgical excision. Neo-adjuvant radiotherapy was delivered by means of a 4-field technique to a dose of 40 Gy in 20 fractions of 2 Gy. Six weeks later a CT-scan, revealed a major downsizing with a maximal diameter of the tumour of 47 mm, which was initially 72 mm (figure 1B). A subsequent laparotomy revealed a mobile tumour and a radical resection was performed without complications. An intraoperative boost (IORT) of 10 Gy was applied to the presacral resection surface. The patient recovered without complications and final histology showed a cystic residual localisation of CD of the HV type with free resection margins. At present, two years and 3 months after resection of the tumour no signs of recurrence have been detected.

## Discussion

This report documents a unique case of a primary irresectable case of the HV unicentric type of CD which was treated with neoadjuvant radiotherapy and subsequent successful radical resection. To our knowledge, there is no report in the literature describing neoadjuvant radiotherapy as part of the treatment of unicentric CD.

The etiology of Castleman's Disease is unknown. However, CD is associated with other disorders such as HIV infection, POEMS syndrome, amyloidosis, renal insufficiency and increased risk of lymphoma [5,9-11]. Therefore specific systemic therapy related to the associated disorder is indicated. There is no consensus yet concerning the most adequate treatment for CD. Surgery is considered to be the most adequate therapy for unicentric cases of CD as it seems to be curative in most of the cases [4,6,12-20]. Various strategies have been described in case of irresectable unicentric CD ranging from primary radiotherapy, incomplete resection [10] and chemotherapy [31].

Primary radiotherapy has been described in numerous case reports and small case series as one of the strategies for the treatment of both unicentric and multicentric forms of CD. Keller et al [4] however described that in 4 cases primary radiotherapy had only a minimal effect and concluded that radiotherapy alone was not effective. However, several cases have been reported with significant responses to primary radiotherapy, for the unicentric as well as for the multicentric form of CD [9,10,21-23,25-30,32,33]. Table 1, presents an overview of all studies that have evaluated the use of primary radiotherapy in CD, both for unicentric and multicentric disease, with doses ranging from 12 to 50 Gy [4,8-10,22-30,32,34,35]. Responses to primary radiotherapy for the treatment of both forms of CD ranged from no response to a complete response. Nevertheless, 88% of all CD patients treated with radiotherapy showed a response, of which 43,8% showed a complete response.

**Table 1:** Summary of reports describing patients with CD treated with primary radiotherapy

Reference	Number of patients (n = 32)	Histopathology	Type	Dose	Response
Fitzpatrick et al. (1968)	1	-	Unicentric	4500	PR
	1	-	Unicentric	-	CR
Keller et al. (1972)	4	HV	Unicentric	1800-4300	PR
Emson et al. (1973)	1	-	-	3500	PR
Nordstrom et al. (1978)	1	PC	Unicentric	2700	PR
Nordstrom et al. (1978)	1	PC	Unicentric	2700	PR
	1	PC	Multicentric	1500	PR
Gaba et al. (1978)	1	HV	Multicentric	4500	NR
Weisenburger et al. (1979)	1	PC	Unicentric	2700	PR
Marti et al. (1983)	1	M	Multicentric	1200	CR
Stokes et al. (1985)	1	PC	Unicentric	3939	PR
Sethi et al (1990)	1	HV	Unicentric	4500	CR
Massey et al. (1991)	1	M	Unicentric	3040	PR
Latz et al. (1992)	1	HV	Unicentric	4000	CR
Veldhuis et al. (1996)	1	PC	Unicentric	4000	CR
Bowne et al. (1999)	1	HV	Unicentric	4500	NR
Chronowski et al.(2001)	4	HV	Unicentric	4000	CR
	4	HV	Unicentric	3960	CR
	2	HV	Unicentric	3960	PR
Neuhof and Debus (2006)	1	HV	Unicentric	4000-5000	NR
	1	HV	Unicentric	4000-5000	CR
Neuhof and Debus (2006)	1	M	Unicentric	4000-5000	NR
All Reports	32				CR: 14/32:43,8% PR: 14/32:43,8% NR: 4/32:12,4%

PC (plasma cell type); HV (hyaline vascular type); M (mixed HV and PC type); PR (partial response);

CR (complete response); NR (non response);

With respect to the dose of radiotherapy, no correlation can be observed between dose and response. Most patients were treated with a dose between 40 and 50 Gy, however patients with a complete response received a dose between 12 Gy and 50 Gy.

The evidence in the current literature reveals that radical surgery results in excellent rates of cure [4,6,12-19]. Also the literature reports excellent response rates with primary radiotherapy (table 1). Therefore it was concluded that, in case of an irresectable presentation of unicentric CD, surgery after neoadjuvant radiotherapy was a possible strategy. This case illustrates that this hypothesis was successful and should be considered as a strategy in case of irresectable unicentric disease. The question remains which policy should be followed in case of a complete radiologic response. Although merely speculative, a wait and see policy with regular radiological follow-up could be a reasonable strategy in case of no systemic symptoms, since most reports describe unicentric CD as a slowly progressive disease, which does not metastasize. In the case where a recurrence of residual disease is found, a resection in an early stage could still be performed. In that case we propose a frequent follow up, using CT scanning every six months, since unicentric CD is a slowly progressive disease. In case of a partial response to primary radiotherapy, we suggest that surgical resection is strongly recommended, since radical surgery can potentially cure unicentric CD.

Surgery was performed six weeks after the last radiotherapy dose. The rationale for this interval is based on the experience gained from treatments with other neoadjuvant strategies in solid tumours [36].

Different types of therapy have been described for the treatment of patients with multicentric CD. However the treatment strategy for this variant of CD is not within the scope of this article. Nevertheless, the literature reveals that primary radiotherapy can also achieve a remission of symptoms [10,24,26,28,30,32] and given the fact that multicentric CD, more often then unicentric CD, causes generalised symptoms, radiotherapy could also be used as a symptomatic treatment.

The rationale of our additional intra-operative radiotherapy (IORT) boost was based on the close relationship with the sacral bone and iliac vessels and was analogous to our management of other locally advanced solid tumours like rectal cancer and soft tissue sarcomas [37-40].

## Conclusion

In this case report it is demonstrated that neo-adjuvant radiotherapy in case of locally advanced irresectable unicentric CD facilitates a radical resection. Therefore, analogous with the treatment of other locally advanced solid tumours, neo-adjuvant radiotherapy with a dose of 40-50 Gy and a subsequent resection 6 weeks later should be considered if an irresectable unicentric variant of CD is encountered.

## Consent

Written informed consent was obtained from the patient for publication of this case report and accompanying images. A copy of the written consent is available for review by the Editor-in-Chief of this journal.

## Competing interests

The authors declare that they have no competing interests.

## Authors' contributions

IAC, MMS, GAP have made substantial contributions to conception and design, and acquisition of data and analysis and interpretation of data. IAC, MMS, T, MLM, JP, GAP have been involved in drafting the manuscript or revising it critically for important intellectual content. All authors read and approved the final manuscript.
